# Retraction note: Down-regulated MicroRNA 148b expression as predictive biomarker and its prognostic significance associated with clinicopathological features in non-small-cell lung cancer patients

**DOI:** 10.1186/s13000-016-0562-7

**Published:** 2016-11-02

**Authors:** Naeemeh Ghasemkhani, Sahar Shadvar, Yasamin Masoudi, Amir Jouya Talaei, Emad Yahaghi, Peyman Karimi Goudarzi, Ebrahim Shakiba

**Affiliations:** 1 Brain and Spinal Injury Research Center, Neuroscience institute, Tehran University of Medical Science, Tehran, Iran; 2 Department of Biophysics, Faculty of Life Science, Azad University of Varamin Pishva Branch, Varamin, Iran; 3 Department of Genetics, Faculty of Life Science, Azad University of Tehran Medical Sciences Branch, Tehran, Iran; 4 Department of Molecular Biology, Baqiyatallah University of Medical Sciences, Tehran, Iran; 5 Department of Neurosurgery, AJA University of Medical Sciences, Tehran, Iran; 6 Department of Biochemistry, Medical School, Kermanshah University of Medical Sciences, Kermanshah, Iran

## Retraction

The Editor-in-Chief and Publisher have retracted this article [1] because the scientific integrity of the content cannot be guaranteed. An investigation by the Publisher found it to be one of a group of articles we have identified as showing evidence suggestive of attempts to subvert the peer review and publication system to inappropriately obtain or allocate authorship. This article showed evidence authorship manipulation.
